# Percutaneous ballon compression for recurrent TN —a retrospective study of 33 cases

**DOI:** 10.3389/fneur.2023.1292804

**Published:** 2023-12-05

**Authors:** Dongwei Feng, Yaxin Zhang, Dong Li, Kang Wang, Fan Yang, Jianan Ding, Weize Wu, Yunhe Wang, Heping Jia

**Affiliations:** ^1^Department of Pain, The First Affiliated Hospital of Hebei North University, Zhangjiakou, Hebei, China; ^2^Department of Breast Surgery, The First Affiliated Hospital of Hebei North University, Zhangjiakou, Hebei, China; ^3^Nuclear magnetic laboratory, The First Affiliated Hospital of Hebei North University, Zhangjiakou, Hebei, China; ^4^Interventional Department, The First Affiliated Hospital of Hebei North University, Zhangjiakou, Hebei, China; ^5^Department of Pathology, The First Affiliated Hospital of Hebei North University, Zhangjiakou, Hebei, China

**Keywords:** percutaneous balloon compression, recurrent, complication, anxiety, pain

## Abstract

**Objective:**

To investigate the clinical efficacy of percutaneous microballoon compression in the treatment of recurrent TN.

**Methods:**

This retrospective study included 33 patients who underwent percutaneous microballoon compression for the treatment of recurrent TN from March 2019 to May 2022. Postoperative pain recurrence and facial numbness were assessed according to the Barrow Neurological Institute (BNI) pain score. Patients’ anxiety and sleep status during follow-up were assessed according to the Self-rating Anxiety Scale (SAS) and Pittsburgh Sleep Quality Index (PSQI).

**Results:**

All patients (33 cases) were followed up for 12–38 months, with an average follow-up time of 23 months. On postoperative day 1, 31 patients (93.9%) reported no pain, and 2 patients were given drug treatment for pain relief, The total efficacy was 93.9%. Moreover, 2 patients (6.1%) reported significant pain relief 2 weeks postoperatively. There are many complications during and after PBC. The incidence of the trigeminocardiac reflex (TCR) during surgery was 100%, and the incidence of facial numbness, masseter muscle weakness, labial herpes and headache was 97, 60.6, 12.1 and 3%. No patient experienced severe facial numbness, hearing impairment, diplopia, injury to cranial nerves, Meningitis, intracranial haemorrhage or keratitis. 1 patient had recurrence of pain at 6 months post-op, which was relieved by oral medication. 81.8% suffered from anxiety and 54.5% had poor sleep quality before surgery. After the period of PBC, SAS and PSQI scores decreased continuously. There were significant improvements in anxiety and sleep status postoperatively compared with preoperatively.

**Conclusion:**

PBC is a safe and effective option for the treatment of recurrent TN. The arduous and demanding nature of the clinical course subjects the patient to severe pain, mental, and physical stress. Thankfully, it significantly improves the symptoms of anxiety, depression, and sleep quality.

## Introduction

Idiopathic trigeminal neuralgia (TN) is a neuropathic pathological pain characterized by sudden arrest of the trigeminal nerve on the face and severe shock-like pain (1). The annual incidence is approximately 26.8/100000 (2). Its incidence is mainly in middle-aged and elderly individuals, and its incidence is increasing year-by-year with the ageing of the population (3) Over the years, microvascular decompression(MVD), radiofrequency thermocoagulation (RF), percutaneous retrogasserian glycerol rhizotomy (PRGR), and gamma knife surgery have all achieved some clinical results in the treatment of TN, but the above modalities have significant limitations (4, 5). TN seriously affects the daily work and life of patients and greatly increases the mental stress and economic burden of patients (6). With the deep understanding of the anatomical shape of Meckel’s cavity and the precise control of the balloon pressure magnitude and compression time, percutaneous balloon compression (PBC) based on the concept of “mechanical compression of the semilunar ganglion” has played an irreplaceable role in the treatment of TN because of its simplicity, effectiveness, low recurrence rate and advantages of reproducible treatment (7). Currently, most studies on PBC for recurrent TN focus mainly on pain relief, while few studies have addressed anxiety and sleep disorders in patients. Therefore, the authors retrospectively analysed the clinical data of 33 patients with recurrent TN treated with puncture balloon compression from March 2019 to May 2020 in the Department of Pain, the First Affiliated Hospital of Hebei North University, to verify the efficacy of PBC in the treatment of recurrent TN and provide a theoretical basis for clinical treatment.

## Materials and methods

### Materials

A total of 33 patients who underwent PBC in our department from March 2019 to May 2022 and who were willing to be followed after their operation were selected as the research subjects. This study was approved and implemented by the Hospital Ethics Committee, and all patients who participated in the study signed informed consent.

The inclusion criteria were as follows: (1) medical history, symptoms and signs, and brain computed tomography (CT) and magnetic resonance imaging (MRI) results that all met the International Headache Society diagnostic criteria (8); (2) poor response to drug therapy or intolerance to adverse drug reactions; (3) BNI Pain Intensity Scale scores of IV, V; and (4) previously failed or relapsed trigeminal vascular decompression, percutaneous radiofrequency thermocoagulation of the trigeminal ganglion, gamma knife radiosurgery, percutaneous balloon compression, or percutaneous glycerol lesioning. Patients were excluded if (1) they had severe systemic diseases, such as heart, lung and kidney, and could not tolerate surgery or (2) they had poor compliance and could not cooperate with treatment and follow-up. (3) TN patients who had bilateral symptoms or were diagnosed with secondary TN caused by diseases such as intracranial space-occupying lesions or multiple sclerosis.

### Surgical technique

After general anaesthesia with endotracheal intubation, heart rate and blood pressure were monitored throughout. The patient was placed in the supine position with the head slightly retroverted, and the position, shape, and size of the foramen ovale were determined under C-arm fluoroscopy. To prevent trigeminal reactions, atropine was administered prophylactically preoperatively to patients with heart rates less than 60 beats per minute. The Hartel anterior approach was used to puncture the foramen ovale, and the entry point was selected at the intersection of the vertical line of the lateral canthus and the extended line of the oral angle. A 14-gauge needle was inserted into the opening of the foramen ovale. The 4F Fogarty catheter was connected to a tee joint and slowly introduced into Meckel’s cavity along the puncture path 0.5 ~ 0.8 mL (adjust the dose of contrast agent according to the size of Meckel’s cavity volume). Gadolinium diamine was injected to inflate the balloon to achieve a shape of a pear and was maintained for 3–5 min (compression time was adjusted appropriately based on patient pain, age, balloon pressure and number of previous surgeries). Then, the balloon catheter was withdrawn, the needle core was inserted, and the puncture needle was withdrawn. Compression was applied to the wound for 3–5 min, followed by the placement of a sterile dressing. Patients were instructed to perform masticatory muscle exercises postoperatively.

### Observation indices and effect evaluation

If the postoperative pain relief was poor, close observation was performed for 2 weeks. During this period, patients were administered carbamazepine or oxcarbazepine only if the pain was intolerable. If pain did not resolve after 2 weeks, further treatment, such as medication or minimally invasive intervention, was administered. Outpatient or telephone follow-up was performed at 24 h, 3 months, 6 months, and 12 months after surgery and then once a year. Pain relief was assessed according to the Barrow Neurologic Institute (BNI) pain score scale (9). A pain score of 0 indicated complete painlessness; a score of 1 indicated occasional mild pain that did not require medication to relieve pain; a score of 1 indicated moderate pain that could be controlled with medication; and a score of 3 indicated uncontrollable pain or ineffectiveness of the drug. The self-rating anxiety scale (SAS) (10) was scored as follows: mild anxiety (50–59 points), moderate anxiety (60–69 points) and severe anxiety (≥ 70 points). The Pittsburgh Sleep Quality Index (PSQI) (11) was scored as follows: PSQI total score (0 ~ 21 points), PSQI total score ≥ 8 points, indicating poor sleep quality.

Complications mainly included facial numbness, masticatory muscle weakness, pain recurrence, perioral herpes, diplopia, keratitis. Among them, facial numbness was assessed using the BNI facial numbness scoring criteria (9): Grade I, no facial numbness; Grade II, mild facial numbness that is bothersome or slightly bothersome in daily life; Grade III, moderate facial numbness that is bothersome in daily life; and Grade IV, severe facial numbness that is extremely bothersome in daily life.

### Statistical analysis

Data analysis was performed using SPSS version 22.0 (IBM Corp, United States) to analyse all data; all variables were examined for normal distribution. The measurement data are expressed as the mean ± standard deviation (SD). The enumeration data are expressed as percentages. Repeated-measures analysis of variance was used to analyse the BNI-P score at different time points. SAS and PSQI scores at different time points were analysed using the nonparametric Cochran Q test. The statistical significance level was set at α = 0.05.

## Result

All patients were followed up for an average of 23 (12–38) months, On postoperative day 1, 31 patients (93.9%) reported no pain, and 2 patients were given drug treatment for pain relief, The total efficacy was 93.9%. Moreover, 2 patients (6.1%) reported significant pain relief 2 weeks postoperatively. The details are shown in Table 1.

**Table 1:** The demographic characteristics of 33 patients.

Male	11 (56%)
Female	22 (44%)
Age	71 (29 ~ 86)
Trigeminal division	
I	2
II	13
III	4
I + II	2
II + III	8
I + III	2
I + II + III	2
Symptom duration (years)	5(2 ~ 20)
Distribution by side	
Left side	20
Right side	13
Previous failed procedures	8
Microvascular decompression	2
Radiofrequency thermorhizotomy	10
Percutaneous balloon compression	12
Glycerol gangliolysis	1
Time since last surgery	37(12 ~ 80)
Follow-up time (months)	23(12 ~ 38)

There are many complications during and after PBC. The incidence of the trigeminocardiac reflex (TCR) during surgery was 100%, Giving anticholinergic drugs before surgery may be a way to prevent TCR. There were 32 cases (97%) of facial numbness (22 cases of BNI numbness grade II and 10 cases of grade III), and the BNI numbness grade was II or less at 12 months after surgery. 20 cases (61%) developed varying degrees of masticatory muscle weakness, which gradually recovered after 2 to 10 months. 4 patients (12%) developed perioral herpes simplex and were cured after treatment with antiviral drugs. 1 patient developed ocular discomfort, which improved after 2 weeks. There was 1 case (3%) recurrence at 6 months after surgery. Taking into account economic factors, the patients were tolerant to pain after taking medication and did not undergo repeated PBC. No patient experienced severe facial numbness, hearing impairment, diplopia, injury to cranial nerves, Meningitis, intracranial haemorrhage or keratitis. The details are shown in Table 2.

**Table 2:** The complications of the 33 patients during/after PBC.

Inhibitory reactions of the trigeminal	33
Facial numbness	32
Masseter muscle weakness	20
Labial herpes	4
Keratitis	0
Diplopia	0
Headache	1
Injury to cranial nerves	0
Meningitis	0
Arterial hemorrhage	0

BNI-P scores of TN were significantly lower at 1 day post-operative and 1-, 3-, 6-, and 12-month follow-up compared with pre-surgery (*p* < 0.001). The details are shown in Table 3.

**Table 3:** Outcomes of the observation indexes before and after PBC treatment.

SAS				PSQI	
Normal	Mild	Moderate	Severe	PSQI<8	PSQI≥8
0	6	15	12	15	18
0	21	10	2	29	4
9	15	8	1	31	2
14	14	4	1	30	3
25	5	1	2	29	4
*Z* = 76.538				*Q* = 53.576	
*p* < 0.001				*p* < 0.001	

Comparison of anxiety scores in different periods, analyzed by rank sum test, *Z* = 76.538, *p* < 0.001. Comparison of sleep scores at different stages, analyzed by Cochran ‘s Q test, *Q* = 53.576, *p* < 0.001.

The SAS and PSQI scores significantly decreased in different at 1 day post-operative and 1-, 3-, 6-, and 12-month follow-up compared with pre-surgery (p < 0.001). The details are shown in Table 4.

**Table 4:** Outcome of Pain indexes before and after PBC treatment.

Period	BIN-P
Preoperative	3.00
24 h post-op	0.18 ± 0.53
3 months post-op	0.12 ± 0.42
6 months post-op	0.15 ± 0.44
12 months post-op	0.21 ± 0.60
Statistical value	*F* = 355.04
*p*	*p* < 0.001

Comparison of pain at different time periods, analyzed by repeated measures variance test, *F* = 355.04, *p* < 0.001.

## Discussion

At present, there is no uniform opinion on the treatment of recurrent TN (12). Surgical treatment options for TN include MVD, PBC, RF, PRGR and gamma knife surgery (13). MVD is the first choice for vascular pressure pressors, but this procedure is invasive and thus not recommended in older patients with poor general condition (14). RF is a safe and effective method for the treatment of TN, but it is not suitable for those with multivessel trigeminal nerve involvement (15). Yang et al. (16) used gamma knife radiosurgery to treat 16 patients who failed to respond to gamma knife radiosurgery or relapsed, of whom the effective rate of the first gamma knife effective retreatment was 90% (9/10); however, the effective retreatment rate of the patients with previously ineffective gamma knife radiosurgery was 33% (2/6), and its final definitive effect was often unpredictable. PRGR is injected into Meckel’s cave to damage the nerve fibres that cause pain (17). Facial hypoesthesia and corneal reflex are more affected with PRGR than with PBC, and PRGR requires patients to be awake; thus, there are many factors that may affect the effectiveness of this operation (18).

PBC was first reported by Professor Mullan in 1983. It is a method to relieve pain by mechanical compression of sensory fibres in TN using a microballoon (19). Compared with many minimally invasive or noninvasive therapies, PBC has a simple overall surgical procedure, short operation time, small surgical wounds, and repeatable procedures (20). Furthermore, the postoperative complications of PBC do not affect the daily life of patients. As a result, PBC has been increasingly used by clinicians in recent years (21). Ying et al. (22) used PBC to treat 138 patients, and the pain cure rate was 98.6% at 24 h after surgery, and a low recurrence rate was maintained within 3, 4, and 5 years after surgery. The authors concluded that PBC is a safe and effective method. Another retrospective analysis (7) on 121 patients with recurrent TN treated with PBC showed that the effective rates of pain at 24 h, 1 month, 3 months, 6 months, and 12 months after surgery were 93.4, 95.9, 95.9, 94.2, and 91.7%. However, few studies have addressed the efficacy of PBC for recurrent TN.

For this study, all 33 patients had recurrent TN, we observed a total efficacy of 93.9%, And BNI-P scores of TN were significantly lower at 1 day post-operative and 1-, 3-, 6-, and 12-month follow-up compared with pre-surgery (*p* < 0.001) this is also similar to previous studiesand. At the same time, we found a recurrence rate of 1%, which is lower than previous studies. This may be associated with improvements in surgical techniques. Correct needle insertion at the foramen ovale and balloon insertion in Meckel’s lumen are two important steps in PBC (23). Using imaging techniques, we can determine if the needle accurately reaches the foramen ovale and is in the correct position. Balloon shape can also be visualized by imaging. When the balloon does not form a pear shape, the contrast agent is withdrawn immediately and the needle is repositioned until the balloon is in a typical pear shape. This is an important imaging indication to ensure the efficacy of PBC, and balloon pressure and compression time are important factors affecting the postoperative outcome (24). However, there is no uniform standard for balloon pressure and balloon compression time (25), which still needs to be explored.

TN is often accompanied by depression, anxiety, and sleep disorders, which seriously affect the quality of life of patients, especially those with recurrent disease (12). Zakrzewska et al. (26) found that the incidence of depression and anxiety in patients with TN was 36 and 50%, respectively, in a retrospective analysis of patients with TN but did not further analyse the relief of depression and anxiety at different stages. In this study, the incidence of preoperative severe anxiety and sleep disorders was 81.8 and 54.5%, respectively, which was higher than that in previous studies, which may be related to the this group of patients has a long history. However, the incidence of moderate-to-severe anxiety and sleep disorders decreased to 9.1 and 12.1% during follow-up. There were significant differences in SAS and PSQI scores at different periods compared with pre-surgery (*p* < 0.001). Although most patients had different degrees of depression and sleep disorders before surgery, but the last follow-up showed that SAS and PSQI grades tended to be normal. We reasonably speculated that PBC treatment of recurrent TN can not only effectively relieve pain but also significantly improve anxiety and sleep disorders.

Although PBC is widely used to treat TN, some unavoidable complications that may affect the final treatment outcome have also been reported (27). In this study, the incidence of TCR (trigeminocardiac reflex) during the operation was 100%. Lv et al. (28) found that the incidence of TCR was 97.3%. TCR is essentially a complex brainstem reflex, and any stimulation involving the sensory branch of the trigeminal nerve may induce TCR (29). Bradycardia and blood pressure changes are the most common manifestations of TCR. Thus, TCR may have catastrophic consequences for patients with prior cardiovascular disease. Reasonable prevention generally does not affect the completion of the treatment process. Furthermore, the appearance of this reflex suggests that the balloon compresses the trigeminal ganglion located in Merkel’s cavity, which is a common phenomenon to ensure successful surgery.

Our experience is that once severe cardiovascular changes occur during surgery, the procedure should be stopped immediately, and atropine should be administered intravenously to suppress vagal reflexes. When anti-vagal drugs are not effective, intravenous isoproterenol is infused to ensure that the heart rate is maintained for more than 90 beats. In addition, reducing traction during surgery can reduce the occurrence of TCR responses.

Facial numbness was the most common postoperative complication in 32 patients (97%) in this study. According to Barrow Neurology Institute, facial numbness was classified as Grade IV according to severity (8), according to the above classification, 22 patients were Grade II and 10 patients were Grade III 1 day after surgery, but the BNI numbness was Grade II and below at 12 months after surgery. Facial numbness is closely related to the time of mechanical compression of the semilunar node of the trigeminal nerve by the balloon (30). All patients had recurrence of TN in our study, so we increased the pressure of the balloon as well as the compression time as appropriate, which may be the main reason for the higher incidence of facial numbness. In this study, the rate of masseter muscle weakness, labial herpes, headache were 60.6, 12.1, 3%, which were similar to the above systematic review. However, 1 patient (3%) had recurrence 6 months after surgery. Considering economic factors, the patient was tolerating pain after taking the drug and did not undergo repeat PBC.

This study is affected by the nature of the retrospective study, sample size, systemic factors of patients and personalized treatment, but the preliminary analysis of the treatment results has been roughly analysed, and the relevant influencing factors have not been more meticulously explored. Multicentre, large-sample size, high-quality randomized controlled studies are needed for confirmation.

In conclusion, PBC is a safe and effective treatment for recurrent PBC. PBC treatment of recurrent TN not only has a significant analgesic effect but also significantly improves the anxiety and sleep disorders of patients. The rich experience of surgeons and meticulous intraoperative technique help to reduce the related complications and improve the clinical efficacy.

## Data availability statement

The datasets presented in this study can be found in online repositories. The names of the repository/repositories and accession number(s) can be found in the article/supplementary material.

## Ethics statement

The studies involving humans were approved by The First Affiliated Hospital of Hebei North University. The studies were conducted in accordance with the local legislation and institutional requirements. The participants provided their written informed consent to participate in this study. Written informed consent was obtained from the individual(s) for the publication of any potentially identifiable images or data included in this article.

## Author contributions

DF: Writing – original draft. YZ: Conceptualization, Data curation, Investigation, Methodology, Writing – original draft, Writing – review & editing. DL: Writing – original draft, Writing – review & editing. KW: Writing – review & editing. FY: Writing – original draft. JD: Writing – original draft, Writing – review & editing. WW: Writing – original draft, Writing – review & editing. YW: Writing – original draft, Writing – review & editing. HJ: Writing – original draft, Writing – review & editing.
